# The Potential Effects of the Ketogenic Diet in the Prevention and Co-Treatment of Stress, Anxiety, Depression, Schizophrenia, and Bipolar Disorder: From the Basic Research to the Clinical Practice

**DOI:** 10.3390/nu16111546

**Published:** 2024-05-21

**Authors:** Maria Chrysafi, Constantina Jacovides, Sousana K. Papadopoulou, Evmorfia Psara, Theophanis Vorvolakos, Marina Antonopoulou, Antonios Dakanalis, Mato Martin, Gavriela Voulgaridou, Agathi Pritsa, Maria Mentzelou, Constantinos Giaginis

**Affiliations:** 1 Department of Food Science and Nutrition, School of Environment, University of the Aegean, 81400 Lemnos, Greece; fnsm22030@fns.aegean.gr (M.C.); con.jacovides@gmail.com (C.J.); fnsd21013@fns.aegean.gr (E.P.); mk.antonopoulou@yahoo.com (M.A.); fns20084@fns.aegean.gr (M.M.); fnsd22007@fns.aegean.gr (M.M.); 2 Department of Nutritional Sciences and Dietetics, Faculty of Health Sciences, International Hellenic University, 57400 Thessaloniki, Greece; souzpapa@gmail.com (S.K.P.); gabivoulg@gmail.com (G.V.); agpritsa@ihu.gr (A.P.); 3 Department of Psychiatry, School of Medicine, Democritus University of Thrace, 68100 Alexandroupolis, Greece; tvorvola@med.duth.gr; 4 Department of Mental Health, Fondazione IRCCS San Gerardo dei Tintori, Via G.B. Pergolesi 33, 20900 Monza, Italy; antonios.dakanalis@unimib.it; 5 Department of Medicine and Surgery, University of Milan Bicocca, Via Cadore 38, 20900 Monza, Italy

**Keywords:** ketogenic diet, depression, anxiety, stress, schizophrenia, bipolar disorder, animal studies, clinical human studies, psychiatric disorders, carbohydrates restriction

## Abstract

Background: The ketogenic diet (KD) has been highly developed in the past for the treatment of epileptic pathological states in children and adults. Recently, the current re-emergence in its popularity mainly focuses on the therapy of cardiometabolic diseases. The KD can also have anti-inflammatory and neuroprotective activities which may be applied to the prevention and/or co-treatment of a diverse range of psychiatric disorders. Purpose: This is a comprehensive literature review that intends to critically collect and scrutinize the pre-existing research basis and clinical data of the potential advantageous impacts of a KD on stress, anxiety, depression, schizophrenia and bipolar disorder. Methods: This literature review was performed to thoroughly represent the existing research in this topic, as well as to find gaps in the international scientific community. In this aspect, we carefully investigated the ultimate scientific web databases, e.g., PubMed, Scopus, and Web of Science, to derive the currently available animal and clinical human surveys by using efficient and representative keywords. Results: Just in recent years, an increasing amount of animal and clinical human surveys have focused on investigating the possible impacts of the KD in the prevention and co-treatment of depression, anxiety, stress, schizophrenia, and bipolar disorder. Pre-existing basic research with animal studies has consistently demonstrated promising results of the KD, showing a propensity to ameliorate symptoms of depression, anxiety, stress, schizophrenia, and bipolar disorder. However, the translation of these findings to clinical settings presents a more complex issue. The majority of the currently available clinical surveys seem to be moderate, usually not controlled, and have mainly assessed the short-term effects of a KD. In addition, some clinical surveys appear to be characterized by enormous dropout rates and significant absence of compliance measurement, as well as an elevated amount of heterogeneity in their methodological design. Conclusions: Although the currently available evidence seems promising, it is highly recommended to accomplish larger, long-term, randomized, double-blind, controlled clinical trials with a prospective design, in order to derive conclusive results as to whether KD could act as a potential preventative factor or even a co-treatment agent against stress, anxiety, depression, schizophrenia, and bipolar disorder. Basic research with animal studies is also recommended to examine the molecular mechanisms of KD against the above psychiatric diseases.

## 1. Introduction

The defining characteristic of a ketogenic diet (KD) is the production of ketones, including acetoacetic acid, β-hydroxybutyric acid, and acetone, which can be considered as an alternative energy basis for the central nervous system and tissues. The KD is distinguished by its elevated fat (65–80% of total energy), modest protein (20–25% of overall energy), and low carbohydrate content (5–10% of total energy), effectively mimicking the metabolic effects of fasting [1,2]. The historical development of KD can be traced back to 1921, when it was first applied for the management and control of drug-resistant epilepsy [3]. Notably, Woodyatt initially observed the production of acetone and β-hydroxybutyric acid in healthy individuals, after fasting or consumption of a very low-carbohydrate, high-fat diet in the same period [2,3,4]. Concurrently, testing of the KD in patients suffering from epilepsy has been proposed, suggesting that ketonemia could be induced through alternative means [2,4].

In its original form, the KD consists of a very restrictive regimen, with the proportion of fat to protein to carbohydrate normally in a ratio of 3:4:1 [2,5,6]. In ongoing medicine, the KD has evolved beyond its original application for epilepsy management and has gained recognition for its potential beneficial effects against a variety of pathological conditions. Notably, various forms of the KD have been developed to address different clinical demands, ranging from body weight management to neurological disorders, while recent research has shed light on the potential cardiovascular benefits of a KD [5,7,8]. Emerging evidence has suggested that the metabolic and neurobiological effects of the KD extend beyond seizure control, offering potential advantages for cardiovascular health, cancer treatment, and inflammation reduction, while also impacting various aspects of brain function and mental well-being [9,10,11,12,13].

The prevalence of mental health illnesses, including stress, anxiety, depressive behavior, schizophrenia, and bipolar disorder, continues to rise globally, posing significant challenges for both individuals and healthcare systems [14]. Current treatment approaches are often reliant on pharmacotherapy, psychotherapy, or a combination of these. Conventional pharmacological interventions, while effective for some patients, often come with undesirable side effects and limitations in efficacy [15,16]. Hence, it is critical to take into consideration the pharmacokinetic and pharmacodynamic interrelationships that could occur concerning KD and diverse medications [17]. As such, there is an urgent demand to explore novel therapeutic modalities that offer improved outcomes and tolerability.

The present review study aims to provide a thorough overview of the potential impacts of the KD in the prevention and co-treatment of the most typical psychiatric diseases in both animals and humans. Pre-clinical studies in animals have provided compelling evidence of the impact of a KD on diverse neurobiological pathways implicated in mood and psychotic disorders, including stress resilience, neurotransmitter systems, neuroinflammation, oxidative stress, and mitochondrial function [18,19,20,21]. By synthesizing evidence from basic research studies elucidating the underlying mechanisms of KD action, to clinical trials evaluating its therapeutic efficacy, we aim to bridge the gap concerning pre-clinical insights and real-world clinical applications.

Notably, despite the promising findings, several gaps in the knowledge remain regarding the specific processes related to the therapeutic impacts of the KD in psychiatric diseases. The present study will explore the practical considerations and challenges associated with implementing the KD in clinical practice, including dietary adherence, monitoring of metabolic parameters, and individualized treatment strategies. By critically evaluating the existing literature and identifying key areas for future research, we aim to contribute to the existing body of knowledge surrounding the role of metabolic interventions in mental health promotion and disease management.

## 2. Methods

A comprehensive and detailed literature review was carried out searching the most reliable scientific databases, e.g., PubMed, Scopus, and Web of Science, applying effective, and relevant keywords such as “ketogenic diet AND depression”, “ketogenic diet AND anxiety”, “ketogenic diet AND stress”, “ketogenic diet AND schizophrenia”, “ketogenic diet AND bipolar disorder”, “ketogenic diet AND mental disorders”, “ketogenic diet AND mood disorders”, “ketogenic diet AND psychiatric diseases”, “ketogenic diet AND neurobiological effects”, “ketone bodies OR ketosis”, “carbohydrates restriction OR calories restriction”, “ketogenic diet AND clinical trial”, “ketogenic diet AND animal studies”, “ketogenic diet AND epidemiological studies”, “ketogenic diet AND β-hydroxybutyrate”, “nutritional ketosis AND β-hydroxybutyrate”, “nutritional intervention AND ketosis”, and “medium-chain triglyceride AND ketogenic diet”. The inclusion criteria were surveys authored in the English language, especially in vivo animal experimental surveys, as well as clinical human studies. Gray literature, editorial texts, commentaries, letters to the editor, abstracts in congress records, and articles in non-peer-reviewed journals were excluded from the final analysis. The search was extended by checking the reference lists of relevant studies and manually searching the reference lists of key journals, commentaries, editorials, and abstracts in congress records. The retrieved studies were carefully searched for relevant surveys included in their manuscript. There was not any time limitation concerning the included studies retrieved by the scientific databases.

## 3. Results

### 3.1. Depression

#### 3.1.1. Animal Studies

The animal studies exploring the possible impacts of the KD on depression are presented in Table 1. A recent study investigated the impacts of a mixture of a KD and repeated voluntary exercise on depressive performance in 32 adult male Balb/c mice [22]. A combination of KD with repeated voluntary exercise decreased depressive performance in rodent animals. The adoption of the KD with repeated exercise increased β-hydroxybutyrate (BHB) levels, and decreased not only glucose and insulin, but also low-density lipoprotein/high-density lipoprotein (LDL/HDL) proportions [22]. The KD increased exercise performance, because of the elevated BHB levels. The above findings supported evidence that elevated BHB levels and reduced glucose, or insulin levels and LDL/HDL proportion, could directly or indirectly contribute to the positive impacts of a KD on depressive symptoms [22].

The possible beneficial impacts of the KD on depressive behavior, applying the repeated social defeat stress (R-SDS) and lipopolysaccharide (LPS) animal models (2~3-month-old male C57BL/6J mice), have also been assessed [23]. Using electrophysiological, genetic, and biochemical laboratory techniques, additional investigation of the possible fundamental mechanisms by which a KD may exert its anti-depressive properties was conducted [23]. More to the point, KD treatment considerably attenuated depressive-like behaviors in both R-SDS and LPS models, demonstrating the possible beneficial impacts of KD on depressive symptomatology. Electrophysiological studies additionally indicated that neuronal excitability was elevated in the lateral habenula (LHb) of mice subjected to R-SDS or LPS exposure, that were upturned by adopting a KD [23]. In particular, the potential beneficial impacts of a KD on depression symptomatology were established. These potential therapeutic effects were, at least in part, ascribed to the restoration of microglial inflammatory activation and neuronal excitability. In addition, the researchers have provided evidence for a previously unknown action of Trem2 in the LHb, related with depression. The above survey provided novel evidence concerning the pathogenetic mechanisms of depression and thus recommends a possible, effective treatment approach [23].

Furthermore, the neurological physiology and activities of 8-week-old young adult CD-1 mice, which received a KD in utero and consumed merely a standard diet (SD) in postnatal life, were assessed [24]. In fact, standardized neuro-behavior tests were used, including the open-field, forced-swim, and exercise wheel assessments. The above neuro-behavior tests were combined with post-mortem magnetic resonance imaging (MRI) to explore brain anatomy. Six-week-old male and female CD-1 mice were weight-paired and randomly enrolled in a control group or an intervention group (KD) [24]. The adult KD offspring exhibited lowered depression sensitivity and elevated physical activity levels compared to controls that received an SD both in utero and postnatally. Several neuro-anatomical alterations were observed between the KD offspring and controls, including for instance, a cerebellar volumetric increase of 4.8%, a hypothalamic decrease of 1.39%, and a corpus callosum decrease of 4.77%, computed relative to the overall brain volume [24].

Another study aimed to analyze the possible effects of KDs, including long-chain and medium-chain fatty acids, on cerebral electrical action, evaluating the propagation of the process of cortical spreading depression (CSD) [25]. For this purpose, three groups of weaned rats (21 days old) were treated for seven weeks with either a control (AIN-93G diet), a medium-chain fatty acids KD (including high levels of triheptanoin oil), or a long-chain fatty acids KD (including high levels of soybean oil). All groups were evaluated in comparison with another three groups (21 days old) treated with similar diets for exactly ten days [25]. CSD propagation was assessed exactly, next to the end of the nutritional interventions. This study revealed that short-term KD intervention may result in a substantial decrease in the CSD velocity of propagation, compared to the control group. However, long-term treatment with both KDs did not show any considerable effect on the CSD velocity [25].

#### 3.1.2. Clinical Studies

The clinical human surveys assessing the possible effects of a KD on depression are presented in Table 2. A recent case report exploring a 68-year-old woman diagnosed with Parkinson’s stage I disease with moderate depressive symptomatology was performed [34]. The women received a conventional KD (fats 70%, protein 25%, carbohydrates 5%) for a period of 24 weeks. Low levels of improvement were observed on depressive scale scores at 24 weeks, as measured by the Center for Epidemiologic Studies Depression-Revised (CESDR-R-20) scale [34]. Moreover, a small pilot survey including 16 adult individuals from age 36–80 years old who were diagnosed with Parkinson’s disease participated in a nutritional intervention for 12 weeks [35]. As nutritional intervention, a low carbohydrate/healthy fat/KD (LCHF/KD) was received by the enrolled patients to assess any impacts of the LCHF/KD on depressive symptomatology, as determined by the commonly utilized CESD-R-20 scale. This study did not find any considerable alterations in self-reported depressive symptomatology scores after 12 weeks of the intervention [35].

In a retrospective clinical study, 31 adults with severe, persistent mental disease (major depressive disease, bipolar disease, and schizoaffective disease), whose symptoms were monitored less in spite of thorough psychiatric managing, were admitted to a psychiatric clinic [36]. The enrolled patients received a KD limited to a maximum of 20 g of carbohydrate daily, complementary to standard inpatient care [36]. The period of adopting the KD was between 6 and 248 days. Amongst assigned individuals, means and standard deviations were considerably better, based on both the Hamilton Depression Rating Scale scores from 25.4 (6.3) to 7.7 (4.2), and the Montgomery-Åsberg Depression Rating Scale scores from 29.6 (7.8) to 10.1 (6.5) [36]. Another non-randomized, open-label clinical study enrolled 262 primarily non-depressed diabetes type II patients from ages 21–65 years and with a body mass index (BMI) of >25 kg/m^2^ [37]. This study explored potential differences in depression symptomatology across two years [37]. More to the point, depressive symptoms were determined throughout individuals’ clinic visits, utilizing the CESD-20 scale. Subclinical depression symptomatology reduced during the initial ten weeks and the decreases were retained for at least two years. In addition, enhanced incidence of blood ketone concentrations, representative of compliance to low carbohydrate consumption, was able to predict reductions in depressive symptoms [37]. There are several concerns that were recognized regarding the proposed limiting nutritional patterns, because of the probable undesirable effects on quality-of-life factors, such as mood. Nevertheless, this clinical study seemed to exert beneficial rather than harmful long-term effects of strictly monitored carbohydrate restriction on depression symptomatology [37].

In another study, 93 overweight or obese participants were randomly assigned to an energy-restricted, planned isocaloric LCHF diet or a high-CLF (HCLF) diet for eight weeks [38]. Body weight and psychological well-being were assessed by utilizing the Profile of Mood States (POMS), and the Beck Depression Inventory (BDI) instruments at baseline and fortnightly. This study compared the impacts of a LCHF diet with a typical HCLF diet on mood and cognitive function [38]. Both nutritional interventions resulted in better psychological well-being, with the highest level of effect appearing in the initial two weeks. However, there was not find any considerable difference between the above groups [38]. A later, larger, randomized, controlled clinical survey compared the long-term impacts of a modest energy-restricted low-carbohydrate (LC) diet with those of a typical isocaloric low-fatty (LF) diet on mood and cognitive function in overweight or obese individuals [39]. In fact, 106 individuals who were overweight or obese, with a mean age of 50.0 ± 0.8 years old, were randomly enrolled either to an energy-restricted (about 1433–1672 kcal), planned isocaloric, very low-LCHF diet, or to a HCLF diet, during a period of one year. Then, alterations in body weight, psychological mood, and well-being (Profile of Mood States, Beck Depression Inventory) were assessed [39]. This study showed a considerable impact of diet composition, revealing that scores improved over the longer term, while afterwards they continued to be stable for those individuals enrolled in the LF diet (i.e., a beneficial impact of the diet on mood remained). In contrast, in the LC group, in spite of an initial improvement, the above scores returned towards baseline levels over time (i.e., mood returned toward more harmful baseline levels) [39].

**Table 2:** Clinical studies about KD in mental disorders.

Mental Disorder	Study Type	Study Population	Intervention	Main Results	References
**Depression, anxiety**	Case study	68-year-old woman with PD	Traditional KD for 24 weeks	The adoption of the traditional KD led to minimum improvement on CESD-R-20 scale. Non-significant reduction of PAS score was also observed.	Tidman et al., 2022 [34]
**Depression, anxiety**	Pilot study	16 adults with PD	LCHF/KD for 12 weeks	No significant changes in CESD-R-20 scale were noted.	Tidman et al., 2022 [35]
**Depression**	Non-randomized, open-label study	262 non-depressed T2D adult patients	Carbohydrate restriction that led to nutritional ketosis	Mood improvement and reduction of depressive symptoms, maintained during the two-year intervention.	Adams et al., 2022 [37]
**Depression**	Case report	A 65-year-old female with uncontrolled T2D and MDD	Clinically prescribed KD for 12 weeks	After the intervention, normalization of HgA1c, reduction of glucose levels, and improvement of HOMA-IR and triglyceride/HDL ratios by 75% were observed. The PHQ-9 score dropped to 0.	Cox et al., 2019 [40]
**Depression, schizoaffective disorder, bipolar disorder**	Retrospective analysis	31 adults with severe mental disorder	KD	Significant improvements in the Hamilton Depression Rating Scale and the Montgomery-Åsberg Depression Rating Scale scores were observed after the KD intervention. Moreover, the PANSS scores of the patients with schizoaffective disorder improved significantly.	Danan et al., 2022 [36]
**Mood disorders including depression and anxiety**	Randomized, controlled trial	93 overweight or obese adults	LCHF KD or HCLF diet for eight weeks	Both groups experienced improvements in psychological well-being but with no considerable differences between the two of them.	Halyburton et al., 2007 [38]
**Mood disorders including depression and anxiety**	Randomized, controlled trial	106 overweight or obese adults	Energy-restricted LC KD or isocaloric LF diet for one year	Body weight, Profile of Mood States scores, Beck Depression Inventory, and Spielberger State Anxiety Inventory scores improved and remained stable for the LF group. In the LC group, the scores improved initially but over time returned to baseline levels.	Brinkworth et al., 2009 [39]
**Anxiety**	Case report	37-year-old female patient with panic attacks	Atkins diet	Increased frequency of panic attacks after the adoption of Atkins diet. Improvement of the symptoms that totally resolved after quitting the Atkins diet.	Ehrenreich et al., 2006 [41]
**Sleep anxiety**	Cross-sectional, prospective study	14 children with DRE and their mothers	KD for three months	KD led to significant improvement of sleep anxiety in children with DRE. Better sleep quality was observed in the working mothers as well.	Ünalp et al., 2021 [42]
**Perceived stress, depression and anxiety**	Online cross-sectional study distributed into two cohorts	Cohort 1: Bond-Lader visual analog scales and Perceived Stress Scale (*n* = 147). Cohort 2: Depression Anxiety Stress Scale (*n* = 276).	KD	Stress, depression, and anxiety scores were significantly worse for those adopting a KD than those receiving other diets	Garner et al., 2024 [43]
**Schizophrenia**	Uncontrolled, pilot trial	Ten patients with schizophrenia	KD	Following the KD led to significant reduction the symptoms.	Pacheco et al., 1965 [44]
**Schizophrenia**	Case report	A 70-year-old woman with schizophrenia	KD	When following a KD, there was no relapse of the auditory or visual hallucinations of the patient.	Kraft & Westman, 2009 [45]
**Schizoaffective disorder**	Case study	A 33-year-old man and a 31-year-old woman with schizoaffective disorder	KD	In both patients, the KD resulted in a significant decrease in positive and negative symptoms and in PANSS scores when there was ketosis. These improvements reversed when the ketogenic diet was stopped.	Palmer, 2017 [46]
**Schizophrenia** **and bipolar disorder**	Pilot single arm intervention study	23 outpatients diagnosed with schizophrenia or bipolar disorder	KD for four months	Participants with schizophrenia showed an average 32% improvement according to the brief psychiatric rating scale. The percentage of participants with bipolar disorder who showed >1 point improvement in clinical global impression was 69%.	Sethi et al., 2024 [47]
**Bipolar disorder**	Non-randomized, single-group pilot study	27 euthymic adults with bipolar disorder (20 completed the intervention)	Modified KD for 6–8 weeks	Feasible enrollment and maintenance of euthymic patients with bipolar disorder to a KD intervention. Mild and modifiable adverse effects.	Needham et al., 2023 [48]
**Bipolar disorder**	Case study	A 69-year-old and a 30-year-old woman with type II bipolar disorder	KD	Mood stabilization and significant subjective improvement were observed in both patients, with no adverse effects.	Phelps et al., 2013 [49]
**Bipolar disorder**	Case report	A male patient with bipolar disorder	KD	The KD led to full remission of the disease, reduction of lamotrigine doses and complete discontinuation of quetiapine.	Chmiel et al., 2022 [50]
**Bipolar disorder**	Observational analytical study	274 people with bipolar disorder	KD	141 free-text comments on KDs reported a positive effect on mood stabilization (44.2% had notable mood stabilization and 29.1% had some degree of mood stabilization) or remission of symptoms (12.2%). KDs were also associated with reduced occurrences of depressive episodes, enhanced cognitive clarity and verbal articulation, more energy, and weight loss.	Campbell & Campbell, 2019 [51]
**Bipolar disorder**	Case report	A 49-year-old woman with bipolar disorder	KD	While patient compliance was commendable, ketosis and anticipated clinical improvement due to ketosis were not evident.	Yaroslavsky et al., 2002 [52]

KD: Ketogenic diet, PD: Parkinson’s disease, CESD-R: Center for Epidemiologic Studies Depression Scale-Revised, PAS: Parkinson’s Anxiety Scale, LCHF: Low-carbohydrate high-fat, T2D: Diabetes type 2, MDD: Major depression disorder, LF: Low fat, LC: Low carbohydrates, HOMA-IR: Homeostatic model assessment for insulin resistance, HDL: High-density lipoprotein, PHQ: Patient health questionnaire, DRE: Drug-resistant epilepsy, PANSS: Positive and negative syndrome scale.

A recent case report exploring a 65-year-old woman who had suffered from uncontrolled diabetes type II and persistent major depressive disorder for a period of 26 years has been published [40]. In this case report, the woman with comorbid diabetes type II and major depression symptomatology accomplished a three-month long KD, intended to reduce her glucose levels. The KD intervention was combined with weekly nutrition education, a great intensity period of exercise (corresponded to her cardiovascular conditioning), and an eight 45-min solution-intensive psychotherapy meetings [40]. With the accomplishment of the three-month intervention, considerable outcomes were observed in both physiology and psychology aspects. The woman’s HgA1c levels decreased out of the diabetic range (8.0%) and stabilized at 5.4%. Her mean daily glucose concentrations reduced from 216 mg/dL to 96 mg/dL, while her insulin sensitivity and triglyceride/HDL ratios improved by 75%. Her markers for clinical depression and self-efficacy normalized. This three-month personalized nutritional intervention functioned to considerably inverse the effects of 26 years of diabetes type II, reduced the intensity of two and half decades of chronic depression symptoms, and encouraged the woman with a novel experience of optimism and accomplishment [40].

### 3.2. Anxiety

#### 3.2.1. Animal Studies

The animal experimental surveys assessing the possible effects of a KD on anxiety behaviors are included in Table 1. A recent study investigated the impacts of a combination of KD with conventional intentional physical activity on anxiety-like behavior in 32 adult male Balb/c mice [22]. The intervention resulted in improved anxiety-like behavior in rodent animals. These findings supported evidence that a decline in anxiety-like behaviors could be promoted by a KD and conventional intentional physical activity, which could be related with elevated BHB concentrations and reduced LDL/HDL fraction and insulin and/or glucose concentrations [22]. A previous study also revealed that prenatal exposure to a KD altered neurobehavior in adult mice. In the open field test (OFT), the animals adopting a KD were found to travel smaller distances [24]. In general, smaller distances travelled in the OFT were associated with anxiety behaviors, and this was related with a slower mean speed of motion and a slightly higher period in the center. These findings suggested that mice on a KD had rather lower anxiety symptom intensity, concerning staying to the center of the arena, than the mice with a typical diet. This slight decrease in anxiety could be ascribed to the lowered protein content in the KD group than in the SD group (15.3 vs. 18.9%, respectively) [24].

A survey performed by Calderón et al. showed that rodents adopting a KD showed considerably greater urine concentrations of gamma-aminobutyric acid (GABA) than those receiving a typical diet [26]. These findings provided evidence that KD could be favorable in attenuating anxiety symptoms via its action on GABA. Likewise, mice prenatally exposed to a KD were found to travel smaller distances and to need higher time in the center in an OFT, revealing that a KD could exert considerable effects on anxiety symptoms [26]. Another study determined the impacts of ketone supplements on anxiety symptoms intensity in Sprague-Dawley (SPD) and Wistar Albino Glaxo/Rijswijk (WAG/Rij) rats [27]. The exogenous ketone supplementation was included in food and administrated chronically for 83 days in SPD rats and administered sub-chronically for one week in both rat models, using a daily intragastric gavage bolus followed by the determination of anxiety measures on the elevated plus maze (EPM) [27]. The prolonged use of ketone supplementation with a conventional diet was found to attenuate anxiety symptom intensity in SPD and WAG/Rij rats, as measured by the EPM test [27]. BHB levels were considerably raised, verifying the presence of ketosis [27]. Thus, ketone supplementation could be considered as a hopeful approach to anxiety-related behavior via a new means of stimulating nutritional ketosis.

#### 3.2.2. Clinical Studies

The clinical human surveys determining the possible impacts of a KD on anxiety behaviors are shown in Table 2. A case report examining a 68-year-old woman with Parkinson’s stage I disease and moderate anxiety symptomatology has recently been presented [34]. In fact, the assigned individual adopted a conventional KD (fats 70%, protein 25%, and carbohydrates 5%) for a period of 24 weeks to assess any potential effect of the LCHF/KD on anxiety symptomatology, as evaluated by the Parkinson’s Anxiety Scale (PAS). It was obvious from the woman’s descriptions that there was a decrease in anxiety symptomatology over six months. Nevertheless, the significant level of reduction in the overall scores was not statistically quantifiable [34]. The same research group also performed a small pilot survey, where 16 adults aged 36–80 years with diagnosed Parkinson’s disease participated in an LCHF/KD intervention for a period of 12 weeks [35]. This pilot study revealed substantial alterations in three-month scores concerning PAS, revealing that anxiety symptomatology reduced over the three months. There were also recorded relationships between body weight decline and improved energy levels and self-image, possibly decreasing anxiety and/or depressive symptomatology in certain assigned participating patients [35].

In another study, 93 overweight or obese individuals were randomly assigned to an energy-limited, designed isocaloric LCHF diet or a HCLF diet for two months [38]. This study compared the impacts of an LCHF diet with a typical HCLF diet. There was a significant reduction in Spielberger State Anxiety Inventory (SAI) scores in both groups throughout the intervention, and this effect mostly appeared during the initial two weeks of the nutritional intervention [38]. A large, more recent, randomized controlled survey assessed the long-term impacts of a modest energy-limited LC diet in comparison with those of a typical isocaloric LF diet on mood and cognitive function in overweight or obese persons [39]. Moreover, 106 overweight or obese individuals (mean age, 50.0 ± 0.8 years old) were randomly enrolled either to an energy-limited (about 1433–1672 kcal), designed isocaloric, very LCHF diet, or to a HCLF diet for a period of 12 months. Alterations in body weight were assessed and anxiety levels measured by the Spielberger State Anxiety Inventory. Both groups initially exhibited a decrease in anxiety scores that was of similar magnitude at the eighth week [39]. Nevertheless, over a prolonged term, the alterations in the anxiety scores differed between the two intervention groups. This was ascribed to the fact that the mean scores for the above factors reduced at first in both diet groups, then afterwards showed a trend of staying modest in the HCLF group and, over time, returned to near the initial levels in the very LCHF group [39].

A case report of the re-emergence of panic and anxiety symptoms following the beginning of a high-protein, very LC diet has been reported [41]. The examined patient, aged 37 years old, had a two-week chronicle of daily experiences of elevating panic attacks, including lightheadedness, feeling “sick to her stomach”, chest tightness, faintness, and an overwhelming sense of fear, without depressive symptomatology. The above attacks exhibited sudden onset and continued for a period of 30–45 min [41]. When the patient was 47 years old, she chose to adopt the Atkins diet. One day after adopting the Atkins diet, the patient started to experience an internal feeling of “shakiness”. This ultimately developed into a full-fledged panic attack later that day. The patient then raised her sertraline dosage to 100 mg to attenuate the above symptoms. The patient kept exhibiting repeated panic attacks and there was a considerable elevation in her baseline anxiety symptoms over the following four weeks. She eventually chose to re-start the consumption of carbohydrates and abandon the diet. Her symptoms were gradually attenuated from the first day and all of them were minimized after several days. She remained well over the next years of her life [41].

The potential beneficial effects of KD against anxiety were additionally explored in relation to sleep disturbances, as people with anxiety usually exhibit inadequate sleep quality and insomnia [53]. A survey performed by Hallböök et al. examined 18 children with epilepsy and showed that their sleep quality became considerably better, and their rapid eye movement (REM) was improved, by adopting a KD for an interval of 12 weeks [54]. In agreement with the above evidence, a 12-week KD treatment considerably attenuated the sleep anxiety of children with epilepsy, implying that a KD could exert beneficial impacts on the total sleep quality [42]. Moreover, 11 children persisted to adopt a KD and were evaluated again after one year. The assigned children exhibited a considerable reduction in daytime sleep and an additional improvement in REM sleep [42].

### 3.3. Stress

#### 3.3.1. Animal Studies

The animal studies exploring the possible impacts of a KD on stress behaviors are presented in Table 1. In this aspect, it should be emphasized that animal models appear to be an invaluable tool in the study of various effects and are aimed at their eventual extrapolation to humans, but such extrapolation should be performed carefully. As far as nutrient effects are concerned, it is always a problem if nutritional and metabolic interspecies differences occur. Laboratory rodents seem to be relatively safe for this purpose, as they are omnivorous like humans. Thus, all effects derived from animal studies could quite effectively be extrapolated to humans without reservations. A study by Yamanashi et al. explored the potential anti-depressant and anti-inflammatory properties of BHB in rats subjected to acute and chronic stress, using a chronic unpredictable stress (CUS) rat model [28]. The effects of acute immobilization (IMM) stress and a unique treatment of BHB on hippocampus levels of IL-1β and TNF-α were examined. Notably, repeated BHB administration attenuated the depressive and anxiety-like behaviors induced by CUS. IMM stress triggered an elevation in hippocampal IL-1β levels, which was reduced by a unique pre-treatment of BHB [28]. Although no significant impact was noted on hippocampal TNF-α concentrations following one hour of IMM stress, a unique BHB pre-treatment lowered TNF-α levels in the hippocampus. The above evidence suggests that BHB may confer antidepressant-like impacts by potentially suppressing NLRP3-mediated neuro-inflammation in the hippocampus, proposing BHB as a promising co-therapy agent against stress-related mood disorders [28].

Furthermore, Ryan et al. explored the impact of ketosis on the hypothalamic–pituitary–adrenocortical (HPA) axis in adult male Long-Evans rats and C57BL/6J mice [29]. Their study revealed that male rodents adopting an LCHF KD displayed recognizable indicators of chronic stress, such as elevated basal and stress-induced plasma corticosterone levels, heightened adrenal sensitivity to adrenocorticotropin hormone, enhanced c-Fos immunolabeling in the paraventricular nucleus of the hypothalamus, and thymic atrophy, indicative of prolonged glucocorticoid exposure [29]. Additionally, the acute administration of MCTs to chow-fed male rodents resulted in a rapid increase in HPA axis activity. Notably, mice that were deficient in FGF21, a hepatokine associated with the ketosis state and metabolic stress, exhibited a diminished HPA response to both KD and MCTs. These findings supported evidence that dietary interventions leading to ketosis may activate the HPA axis, and FGF21 may potentially mediate this effect [29].

Another study by Brownlow et al., on male adult Sprague-Dawley rats showed favorable effects of a KD in chronic stress conditions [30]. More specifically, three dietary conditions were examined: KD, ketone-supplemented (KS), or NIH-31 control diet, under both control and prolonged stress settings. Under control conditions, only KD led to considerable metabolic changes and enhanced performance in the new object detection test. Analysis of the HPA axis response showed that rats adopting a KD were retained under ketosis, in spite of the elevated glucose levels, with no observable diet effects on adrenocorticotropic hormone (ACTH) or corticosterone (CORT) concentrations. Both the KD and KS reduced water maze avoidance latencies, with the KD preventing stress-induced deficits and improving probe test performance [30]. Stress-related hippocampal BHB reduction was alleviated by the KD, and both the KD and KS inhibited stress impacts on BDNF concentrations. Elevated mitochondrial enzymes related with ketogenesis were also observed in KD and KS hippocampus samples, and stress affected glucose transporters mainly in the control diet group. These findings highlighted the multifaceted associations between metabolism, behavior, and hippocampal biochemistry, revealing that endogenous ketosis may improve parameters related to energy and cognitive function, and ketone supplements could replicate biochemical impacts with restricted behavioral impact [30].

A more recent study assessed whether KDs could prevent stress-stimulated symptoms of mood disturbances. In this aspect, the behavioral and neuroendocrinal impacts of a KD in male and female Long-Evans rats subjected to three weeks of chronic mild stress (CMS) while adopting a KD versus control chow (CH) were evaluated [31]. Plasma BHB, CORT, and IL-1β were determined later, along with behavioral testing and hypothalamus corticotropin-releasing hormone (CRH) and neuropeptide Y (NPY) mRNA expression. CMS led to body weight decline in the CH groups. On the other hand, rats adopting the KD were tolerant to CMS-stimulated body weight decline. Female rats adopting the KD were protected from CMS-stimulated reductions in plasma CORT and hypothalamus NPY expression. Together, the above findings supported evidence for the preventative capability of KDs against chronic stress, particularly in females [31].

#### 3.3.2. Clinical Studies

Based on the currently available evidence, the KD has not been explored within the context of stress in randomized controlled clinical trials, and there are no other pilot or small-scale clinical human studies, except for a very recent online cross-sectional study performed by Garner et al. [43]. This study explored the potential relationship between adopting a KD and diverse characteristics of mental health, such as calmness, contentedness, awareness, cognitive and emotional stress, depressive behavior, anxiety, and loneliness, in a general healthy population [43]. Notably, a KD considerably decreased perceived stress scores compared to other diets, and this difference continued to be significant after adjusting for several confounding factors, such as age, BMI, blood pressure, residency, and ownership of accommodation [43].

### 3.4. Schizophrenia

#### 3.4.1. Animal Studies

The animal studies examining the possible impacts of a KD on schizophrenia are included in Table 1. According to Sarnyai and colleagues, the KD can lead to a thorough reinstatement of regular behavioral phenotypes in mice models of schizophrenia [55]. More to the point, while convincing findings help classify brain bioenergetic defects as a cause of schizophrenia and other psychotic diseases, the KD seems to stabilize brain energy metabolism by circumventing glycolysis and relying on ketosis to re-establish regular brain glucose metabolism and mitochondrial function [56,57]. In addition, by permitting the body to depend on ketone bodies as alternate energy substrates, the KD has been considered to alter the GABA/glutamate proportion in favor of GABA in a manner that rewards the decreased GABA concentrations presented in mice with schizophrenia by inhibiting its catabolism and activating its synthesis [55,58].

Kraeuter et al. treated male C57BL/6 mice with the KD for three weeks and induced acute N-methyl-D-aspartate (NMDA) receptor hypofunction by MK-801 (dizocilpine) administration to model the hypo-glutamatergic conditions, which have been assumed to contribute to schizophrenia [32]. Psychomotor hyperactivity and stereotyped behaviors, social withdrawal, and working recall deficits, indicating the beneficial, harmful, and cognitive symptomatology of schizophrenia, respectively, were determined. Notably, mice on a KD exhibited considerably elevated levels of BHB and lowered glucose concentrations compared to mice on an SD [32]. The KD was also found to attenuate the low-dose MK-801-stimulated hyperactivity, stereotyped behaviors, and ataxia. The social relations and spatial working recall disturbances induced by MK-801 were normalized by the KD. The above evidence reinforces that KDs could regularize pathologic behaviors in this animal model of schizophrenia [32]. This proof-of-concept survey did not promptly explore possible mechanisms of KD activity in schizophrenia. Nevertheless, it has been well-recognized that abnormal glutamate neurotransmission, GABA hypofunction, and severe disruptions of glucose metabolism could be implicated in the pathophysiological mechanisms of schizophrenia [32].

Novel transcriptomic, proteomic, and metabolomics surveys have emphasized an abnormal cerebral glucose and energy metabolism as one of the possible pathophysiological mechanisms governing schizophrenia [59]. In this aspect, a substantial study evaluated whether the potential advantageous impacts of a KD can be generalized to diminished pre-pulse inhibition of startle (PPI), a translationally authorized endophenotype of schizophrenia, in a pharmacological model in mice [33]. This study also explored whether the impact of a KD could be related with the calorie-limited condition, which is representative of the primary period of the KD [33]. For this purpose, male C57BL/6 mice received a KD for a period of seven weeks and examined PPI at the third and seventh weeks, in the presence and absence of a meaningful digestible energy deficit, respectively [33]. Notably, KD efficiently inhibited MK-801-stimulated PPI damages at both the third and seventh weeks, irrespective of the presence or absence of digestible energy deficit. In addition, there was no association found between PPI and body weight alterations. The above findings provide supportive evidence for the therapeutic efficiency of the KD in a translational model of schizophrenia, also providing evidence against the impact of calorie limitation in its mechanism of action [33].

#### 3.4.2. Clinical Studies

The clinical human surveys exploring the possible impacts of KDs on schizophrenia are shown in Table 2. In a cross-sectional clinical study, 31 adults with severe, persistent mental disease (major depression disease, bipolar disorder, and schizoaffective disorder), whose symptoms were to a lesser extent controlled, in spite of an intensive psychiatric management, were introduced to a psychiatry hospital and employed on a KD limited to a maximum of 20 g of carbohydrates daily, complementary to typical inpatient care [36]. The period of the interventional study was between 6 and 248 days. Among the ten individuals diagnosed with schizoaffective disease, the average value of the Positive and Negative Syndrome Scale (PANSS) scores significantly improved from 91.4 ± 15.3 to 49.3 ± 6.9 [36].

A no-controlled pilot clinical study of a KD in ten schizophrenia patients documented a constant, considerable attenuation of disease symptomatology for some weeks while on the diet [44]. A 70-year-old woman with chronic disease since her adolescence was shown to experience reduction of all symptoms and interruption of hallucinations after adopting a KD for 12 years [45]. She was capable of discontinuing drug treatment and managed to live without the requirement of a care personnel [45]. In another case report, a 33-year-old man diagnosed with major depressive disease and schizoaffective illness received various drugs, such as lamotrigine and lorazepam, and later adopted a KD for three weeks [46]. Two observations were recognized. Firstly, there was a considerable decrease in body weight (~15 lb). Secondly, there was a substantial improvement in mood, behavior, and an attenuation in schizophrenia-associated symptomatology, including hallucinations [46]. Analogous outcomes were noted in another 31-year-old woman diagnosed with schizoaffective disease and on prescribed medications [46]. Nevertheless, in the last case, the interruption of the KD resulted in a serious symptom recurrence, and after starting the KD again, the symptoms began to expire during a small interval [46].

More recently, a 16-week small clinical survey was performed to examine the effects of a KD on people diagnosed with schizophrenia, presenting metabolic abnormalities also [47]. More to the point, five participants diagnosed with schizophrenia were assigned in a single-arm clinical trial. The individuals with schizophrenia were characterized by a 32% decrease in Brief Psychiatric Rating Scale scores [47].

### 3.5. Bipolar Disorder

#### 3.5.1. Animal Studies

Based on the currently available research evidence, there are not yet any in vivo animal studies assessing the possible impacts of KDs on bipolar disorder. The absence of in vivo animal studies in the international scientific literature has been ascribed to the fact that animal models of bipolar disorder have so far failed to include all the pathophysiological phases of the disease. Thus, limited chosen symptoms have been presented, resulting in the reduced validity of the animal models [60].

Mechanistically, diminished glucose metabolism and mitochondrial dysfunction in the brain seem to constitute the crucial concerns at the basis of bipolar disorder [61]. Experimental data concerning the impact of mitochondrial dysfunction in bipolar disorder have been raised over approximately 20 years of research [62,63]. Both animal models and human individuals have shown enhanced mitochondrial biogenesis, mass, and energy production after adopting the KD [64]. Moreover, significant insulin resistance and blood–brain barrier dysfunction were found to occur in conjunction with bipolar disorder and this has been associated with disease severity, independent of medication status [65]. Pre-clinical surveys using animal models or cell cultures should be performed to confirm the above molecular mechanisms of the action of KDs against bipolar disorder.

#### 3.5.2. Clinical Studies

The clinical human surveys assessing the possible impacts of KD on bipolar disorder are included in Table 2. Novel data from case report studies have suggested that KDs could be efficient for the attenuation of bipolar disorder symptoms. In this aspect, a recent pilot clinical study assessed the compliance and feasibility of adopting a KD in bipolar disorder [48]. This was a single-group non-randomized interventional small clinical survey with no control arm in which euthymic persons with bipolar disorder were enrolled to a 6–8-weeks period of adopting a modified KD. The enrollment and retention of euthymic individuals with bipolar disorder to the 6–8-weeks KD intervention was sufficient, with great compliance rates for outcome measures. Most of the assigned patients obtained and remained ketosis, while the side effects were mostly minor and modifiable [48].

In a case-report study, two women with type II bipolar disorder were capable of maintaining ketosis for long intervals (two and three years, respectively) [49]. Both women showed mood stabilization, which was greater than that obtained with medications. In addition, both women showed a considerable subjective improvement, which was directly associated with ketosis [49]. These two cases revealed that KDs could be considered as a potential sustainable strategy for mood stabilization in cases of bipolar disorder type II. Additionally, they provided evidence that acidic plasma may stabilize mood, probably by lowering intracellular sodium and calcium [49]. In a case report of a male patient, adopting a KD led to full remission of the disorder, while dosages of lamotrigine were reduced, and quetiapine treatment was fully interrupted [50]. Formerly, neither lamotrigine monotherapy nor co-treatment with quetiapine led to euthymia. The impacts of KDs could at least partially be ascribed to the influence on ionic channels and raise in blood acidity (equally to mood stabilizers), the elevation in GABA levels, the regulation of GABA type A receptors, and the inhibition of α-amino-3-hydroxy-5-methyl-4-isoxazolepropionic acid (AMPA) receptors by medium-chain fatty acids [50]. Thus, a KD affected glutamate metabolism and nerve cell metabolism which utilized ketone bodies as energy sources [50]. In contrast, a case-report including one person diagnosed with bipolar disorder did not show any beneficial effect of KD adherence, revealing that ketosis was not obtained [52].

In a small retrospective clinical study, 12 patients diagnosed with bipolar disorder type II were hosted in a psychiatric hospital and were subjected to a KD limited to a maximum of 20 g of carbohydrates daily, complementary to typical inpatient care [36]. The interventional period was between 6 and 248 days. Adopting a KD led to an improvement of several metabolic health parameters; decreasing body weight, BMI, blood pressure, triglycerides, and LDL levels. The KD was related to considerable improvements in depression and psychosis symptomatology [36]. Moreover, an online observational analytic study exploring mood effects of nutritional interventions (ketogenic, omega-3 enriched, or vegetarian diets) categorized by a priori classes of alteration in mood stabilization in 274 individuals diagnosed with bipolar disorder was performed [51]. Considerable mood stabilization, or remission of symptoms over a period, was significantly greater for those on a KD, compared to the other diets. Many participants showed improvements, lasting for prolonged intervals from months to years. Other stated relationships included less symptoms of depression, improved clarity of thought and speech, enhanced energy, and body weight decline [51]. Regardless of the reasonable restrictions of the observational evidence derived from self-reports mailed online, the strong indications concerning the constant benefits reinforced the assumption that KDs may be associated with positive effects on mood stabilization [51]. However, caution should be taken when interpreting the above evidence, since a well-designed clinical study constitutes a prerequisite to establish this assumption. The above primary findings seem to agree with the mitochondrial dysfunction aspect of the bipolar disorder etiology, in which ketone bodies can bypass the blockage between glycolysis and the Krebs cycle [51].

More recently, a four-month small clinical survey was performed to examine the effects of a KD on people with bipolar disorder, also presenting with metabolism abnormalities [47]. The percentage of participants with bipolar who showed >1 point improvement in clinical global impression (CGI) was 69%. Among the enrolled individuals adopting the KD, all of them were in the recovered or recovering status at the completion of the survey. From the 16 bipolar disorder patients, six were primarily recovered and seven recovered before the completion of the survey (six adherent, two semi-adherent) [47]. Just three bipolar disorder patients did not recover, as determined by Clinical Mood Monitoring Forms (CMFs) (one non-compliant, two semi-adherent). Among bipolar disorder patients with baseline symptomatology of at least moderate disease or higher symptom intensity, 79% of them obtained significant improved signs, as assessed by one-point CGI improvement. Amongst subpopulations classified by the compliance rate, significantly improved signs were noted in 88% of the adherent individuals and 60% of the semi-adherent individuals [47].

Mechanistically, the PI3K/AKT/HIF1-a pathway constitutes a potential mechanism connecting insulin resistance and the Warburg effect in bipolar disorder [66]. Downstream impacts of the Warburg phenomenon have also been noted in bipolar disorder [67]. Isocitrate dehydrogenase and α-ketoglutarate dehydrogenase intermediates have been shown to be enhanced in the cerebrospinal fluid of bipolar disorder patients, alongside pyruvate and lactate, revealing a Warburg effect state and an efficient reductive carboxylation [67]. A crucial indicator representing the incidence of reductive carboxylation constitutes the decreased amount of N-acetylaspartate, which can remove the aspartate made in changing glutamate to alpha-ketoglutarate [67]. In drug-naïve individuals with bipolar disorder, lowered N-acetylaspartate has been noted [68]. The second most important sign of reductive carboxylation in traumatic brain injury is the decreased amounts of glutamine derivative citrulline. The above indicator has also been noted in individuals with non-medicated bipolar disorder [69].

## 4. Discussion

The comparative analysis of KD interventions across various mental health disorders reveals both similarities and differences in their therapeutic efficacy, highlighting the nuanced nature of these conditions and the potential for KDs to be used as an adjunctive treatment strategy. Across different disorders, animal studies have consistently demonstrated promising results, with KDs showing a propensity to ameliorate symptoms of depression, anxiety, stress, schizophrenia, and bipolar disorder. However, the translation of these findings into clinical settings represents a more complex picture.

In the case of depression, while animal studies have indicated a possible beneficial effect of KDs against depression-like behaviors, the clinical evidence currently available remains mixed. Animal studies, such as those conducted by Gumus et al. and Guan et al., demonstrated a reduction in depression-like behavior in rodent models following a KD intervention [22,23]. These effects may be mediated by changes in BHB levels and glucose metabolism, as indicated by the observed alterations in certain biochemical markers [22,23]. In addition, Sussman et al. have provided evidence for a potential long-term protective effect of KD exposure in utero against depression in adulthood [24], while the research of de Almeida Rabello Oliviera et al. could not support long-term efficacy of KDs in cortical spreading depression in young rats [25]. In clinical studies, mixed results have been observed so far regarding the impact of KDs on depressive symptoms. While some studies have reported significant improvements [36,37,40], others have found minimal effects or no changes [34,35,38,39]. These discrepancies may be attributed to variations in study design, sample size, duration of intervention, participant characteristics, and the heterogeneity of depressive disorders. Moreover, the lack of long-term follow-up in many clinical studies limits our knowledge of the sustained impacts of KDs on depression. Hence, further large-scale, randomized, controlled clinical trials are warranted to elucidate the potential of KDs as an effective treatment option for depression. In this aspect, a novel randomized controlled clinical trial has recently been designed to explore the efficiency of a six-week program of weekly dietitian advising, plus providing KD meals, compared with an intervention that includes analogous dietetic contact time and encourages a healthy dietary pattern, with elevated vegetable intake and decrease in saturated fat, plus food vouchers to buy healthier items [70]. This clinical study aims to investigate whether a KD could be an efficient intervention to decrease the intensity of depressive symptoms and anxiety, and improve the quality of life and functioning capability of individuals with treatment-tolerant depression [70].

Similarly, as far as anxiety disorders as concerned, animal studies have suggested that KDs may have anxiolytic effects, as evidenced by reduced anxiety-like behaviors in rodents [22,24,26,27]. These findings are consistent with clinical studies reporting reductions in anxiety symptoms following KD interventions [35,38], including sleep disturbances [42,54]. However, individual responses varied and not all studies showed significant improvements [35,39,41]. Variability in study methodologies, including differences in KD protocols and patient characteristics, may contribute to these discrepancies. Moreover, the underlying molecular mechanisms by which a KD can exert its anxiolytic properties remain poorly understood, warranting further investigation. Their anxiolytic properties may be ascribed to their effects on gut microbiome and the improvement of intestinal barrier function [71], as well as the effects of anti-inflammatory properties [72], and the decreased production of reactive oxygen species (ROS) [73].

The interplay between KDs and stress responses unveils a complex relationship, with multifaceted implications for metabolic and neuroendocrine pathways. Yamanashi et al. have demonstrated the possible antidepressant and anti-inflammatory impacts of BHB in rats subjected to acute and chronic stress, suggesting BHB as a promising therapeutic agent for stress-related mood disorders [28]. The study by Ryan et al. [29] focusing on the HPA axis in male rodents maintained on an LCHF KD has revealed indicators of chronic stress, contrasting with the favorable effects observed in the research by Brownlow et al. [30] on Sprague-Dawley rats under chronic stress conditions. While Ryan et al. [29] demonstrated elevated plasma corticosterone levels and heightened adrenal sensitivity in KD-fed rodents, Brownlow et al. [30] showed improved metabolic parameters and cognitive performance in KD-fed rats subjected to chronic stress [29,30]. Moreover, the study by Sahagun et al. [31] on Long-Evans rats explored the potential protective effects of KDs against stress-induced mood disorders, particularly in females. The resistance to CMS-induced body weight loss and maintenance of neuroendocrine markers in KD-fed female rats suggested a gender-specific protective potential of KDs against chronic stress [31]. The above evidence underscores the intricate interrelationships between metabolism, behavior, and neuroendocrine pathways in mediating the effects of KDs on stress resilience. However, further research is strongly recommended to elucidate the underlying molecular mechanisms and validate these findings in human populations.

In the case of schizophrenia, both animal and clinical surveys have suggested that KDs may offer benefits in mitigating symptom severity. Animal models of schizophrenia have consistently shown improvements in cognitive function and reductions in psychotic-like behaviors with KD interventions, possibly through modulation of brain energy metabolism and neurotransmitter balance [32,33,55,58]. Clinical trials have also reported promising findings, with some demonstrating improvements in symptom severity following KD intervention [36,44,45,46]. Nevertheless, the restricted number of clinical surveys and their small sample sizes warrant further investigation.

For bipolar disorder, evidence regarding the efficacy of KD is relatively scarce. Preliminary evidence has suggested that KDs may be beneficial for bipolar disorder, with some case reports, observational studies, and pilot studies reporting improvements in mood stability and symptom severity [48,49,61]. However, larger, well-controlled randomized clinical trials are recommended to establish their effectiveness and safety profile in this population.

Currently, there have been some interesting reviews published that approach the impact of KDs on psychiatric diseases from diverse points of view. A recent narrative review aimed to understand the intricate interrelationship between brain metabolism, sleep, and psychiatric diseases [74]. Another recent review summarized the available research evaluating the effects of the KD on cognition, depression symptoms, and anxiety-associated behaviors, as well as social and dietary behaviors, in laboratory rodents [75]. The majority of the included research in diverse disease animal models shows that the KD may beneficially impact cognitive function. Moreover, an approximately similar number of surveys reported a decreased effect or non-influence of the KD on depressive-associated behaviors. On the other hand, this review study documented that in regard to anxiety-associated behaviors, most of the available surveys did not have any significant impact [75]. A comprehensive overview of preclinical and clinical evidence supporting the use of a KD in the management of mood and anxiety disorders and discussed its relationship with inflammatory processes and potential mechanisms of action for its therapeutic effects [76]. Another review intended to summarize the current knowledge of the sex-specific effects of KDs on mood disorders, from animal models to clinical applications [77].

Our study presents a comprehensive literature review of the possible impacts of the KD on psychiatric disorders, such as depression, anxiety, stress, schizophrenia, and bipolar disorder. A major strength lies in the inclusive approach, incorporating a wide range of animal and clinical studies. By synthesizing evidence from a diverse range of methodologies and populations, we have provided a comprehensive perspective on the therapeutic potential of the KD across various mental health disorders. However, our study has also faced certain limitations. While we aimed for comprehensiveness, the inherent variability in study designs, sample sizes, and methodologies across the included studies, along with limited availability of long-term follow-up data, may introduce bias and affect the robustness of our conclusions. Moreover, while animal studies seem to suggest the promising therapeutic effects of KDs on psychiatric symptoms, translating these findings into human populations is complex, due to interspecies differences in physiology and behavior. Similarly, clinical studies exhibit variability in design, duration, and outcome measures, restricting the generalizability of the currently available results. Despite these challenges, our synthesis highlights the increasingly body of evidence confirming the potential benefits of KDs in psychiatric treatment. Future research should prioritize elucidating the underlying mechanisms of KDs and conducting well-controlled clinical trials to additionally establish the efficacy and safety of the KD in psychiatric care and in personalized treatment approaches.

## 5. Conclusions

In conclusion, while KDs hold promise as a supplementary treatment for mental health disorders, their clinical utility remains to be fully elucidated. Forthcoming research efforts must focus on addressing the methodological limitations of the currently existing studies, exploring the molecular mechanisms implicated in the potential therapeutic impacts of KDs, and optimizing treatment protocols to maximize their efficacy and safety. Collaborative endeavors between researchers, clinicians, and individuals with lived experiences are pivotal to advancing our understanding of the role of KDs in mental health treatment, and improving outcomes for those affected by these challenging conditions.

## Figures and Tables

**Table 1:** Animal studies about KD in mental disorders.

Mental Disorder	Animals	Intervention	Main Results	References
**Depression, anxiety**	32 adult male Balb/c mice	KD with regular voluntary exercise	Increase of BHB, which enhanced exercise performance, along with decrease in glucose, insulin, and LDL/HDL. Decrease in depression-like and anxiety-like behavior in rodents.	Gumus et al., 2022 [22]
**Depression**	Two ~3-month-old male C57BL/6J mice	KD in R-SDS and LPS	KD significantly alleviated depressive-like behaviors in both the R-SDS and LPS models, mediated by restoring microglial inflammatory activation and neuronal excitability.	Guan et al., 2020 [23]
**Depression, anxiety**	8-week-old CD-1 mice 24 on an SD and 20 on a KD	KD in utero	Lower susceptibility to depression in the mice that were exposed to a KD compared to an SD in utero. A 4.8% increase in cerebellar volume, a 1.39% decrease in hypothalamic volume, and a 4.77% decrease in corpus callosum volume, all relative to total brain volume in mice on a KD. The mice on a KD showed slight reductions in anxiety, as indicated by slower average speed and increased duration spent in the center during the OFT.	Sussman et al., 2015 [24]
**Depression**	27 weaned rats (*n* = 8 controls, *n* = 9 MCT-KD, *n* = 10 LCT-KD)	MCT-KD rich in triheptanoin oil and LCT-KD rich in soybean oil	Short-term KDs resulted in a remarkable reduction in the velocity of CSD propagation compared to the control group. Prolonged administration of both KDs showed no discernible effect on the velocity of CSD.	de Almeida Rabello Oliveira et al., 2008 [25]
**Anxiety**	42 male Wistar rats (*n* = 20 controls and *n* = 22 on a KD)	KD for 15 days	Significant increase of GABA in KD-fed rats, which suggests a possible anxiolytic effect of KD through the action of GABA.	Calderón et al., 2017 [26]
**Anxiety**	2-month-old male SPD rats (*n* = 87) and 8-month-old male WAG/Rij rats (*n* = 32)	Ketone supplementation	The chronic and sub-chronic administration of ketone supplements resulted in raised blood BHB concentrations in both animal models, accompanied by a reduction in anxiety-related behavior, as indicated by the results of the elevated plus maze test.	Ari et al., 2016 [27]
**Stress**	64 rats on an SD, divided into controls (*n* = 16), non CUS + PBS) (*n* = 16), CUS + PBS (*n* = 16) and CUS = BHB (*n* = 16)	BHB on CUS and IMM stress	IMM stress triggered an elevation in hippocampal IL-1β levels, which was mitigated by a single pre-treatment with BHB. After 1 h of IMM stress, a single pre-treatment with BHB resulted in reduced TNF-α levels in the hippocampus.	Yamanashi et al., 2017 [28]
**Stress**	Adult male Long-Evans rats, adult male C57BL/6J mice, adult, male FGF21-deficient mice, and wild-type control mice	KD and MCT-KD	Male rodents maintained on a KD displayed indicators of chronic stress. The acute administration of MCTs resulted in a rapid increase in HPA axis activity. Mice deficient in FGF21 exhibited a diminished HPA response to both the KD and MCTs.	Ryan et al., 2018 [29]
**Stress**	60 male adult Sprague-Dawley rats	KD and KS diet under control and chronic stress conditions	A KD significantly enhanced performance in the novel object recognition test under control conditions. Both the KD and KS reduced water maze escape latencies and prevented stress effects on BDNF levels.	Brownlow et al., 2017 [30]
**Stress**	Adult male (*n* = 33) and female (*n* = 37) Long-Evans rats	KD on CMS	KD had a protective effect on CMS-induced weight decline. Female rats adopting a KD were protected from CMS-stimulated reductions in plasma CORT and hypothalamic NPY expression.	Sahagun et al., 2019 [31]
**Schizophrenia**	Male C57BL/6 mice with acute NMDA receptor ypofunction	KD	Considerably elevated BHB and reduced glucose concentrations in mice on a KD compared to the SD. The KD reduced the MK-801-stimulated hyperactivity, stereotyped behavior, and ataxia. Also, it normalized the social relations and spatial working memory damage stimulated by MK-801.	Kraeuter et al., 2015 [32]
**Schizophrenia**	36 male C57BL/6 mice	KD	KD prevented MK-801-induced PPI impairments at both three and seven weeks. A correlation between PPI and body weight changes was not found.	Kraeuter et al., 2019 [33]

KD: Ketogenic diet, BHB: β-Hydroxybutyrate, LDL: Low-density lipoprotein, HDL: High-density lipoprotein, R-SDS: Repeated-social defeat stress, LPS: Lipopolysaccharide, SD: Standard diet, OFT: Open-field test, MCT: Medium-chain triglyceride, LCT: Low-chain triglyceride, CSD: Cortical spreading depression, GABA: Gamma-aminobutyric acid, CUS: Chronic unpredictable stress, PBS: phosphate-buffered saline, IMM: Immobilization, IL-1β: Interelukin-1β, TNF-α: Tumor necrosis factor-α, FGF21: Fibroblast Growth Factor-21, HPA: Hypothalamic-pituitary-adrenal, KS: Ketone supplemented, BDNF: Brain-derived neurotrophic factor, CMS: Chronic mild stress, CORT: Corticosterone, NPY: Neuropeptide Y, PPI: Pre-pulse inhibition of startle.

## Data Availability

The original contributions presented in the study are included in the article, further inquiries can be directed to the corresponding author for private only use.
